# A multicentre validation study of the Swedish version of the Normalization Process Theory Measure S-NoMAD

**DOI:** 10.1186/s43058-025-00839-1

**Published:** 2025-12-15

**Authors:** Anna Cristina Åberg, Lars Wallin, Malin Tistad, Sandra Weineland, Malin Lövgren, Kari Jess, Vilmantas Giedraitis, Johan Lyhagen

**Affiliations:** 1 School of Health and Welfare, Dalarna University, Högskolegatan 2, Falun, S-791 88 Sweden; 2 CIRCLE – Complex Intervention Research in Health and Care, Department of Women’s and Children’s Health, Uppsala University, Box 256, Uppsala, S-751 05 Sweden; 3 Department of Public Health and Caring Science/Geriatrics, Uppsala University, Box 256, Uppsala, S-751 05 Sweden; 4 Department of Psychology, University of Gothenburg, Box 100, Göteborg, S-405 30 Sweden; 5 General Practice/Family Medicine, School of Public Health and Community Medicine, Institute of Medicine, Sahlgrenska Academy, University of Gothenburg, Box 100, Göteborg, S-405 30 Sweden; 6 Department of Health Care Sciences, Palliative Research Centre, Marie Cederschiöld University, Stigbergsgatan 30, Stockholm, S-116 28 Sweden; 7 Advanced Pediatric Home Care, Astrid Lindgren Children’s Hospital, Karolinska University Hospital, Eugeniavägen 23, Stockholm, C12:33, S-171 76 Sweden; 8 Department of Statistics, Uppsala University, Box 256, S-751 05 Uppsala, Sweden

**Keywords:** Implementation measurement, Psychometrics, Normalization Process Theory, Health and social care contexts

## Abstract

**Background:**

The Normalization Process Theory (NPT) is increasingly used for evaluating and understanding implementation processes of complex care interventions. One key tool for applying the NPT in research and practice is the NoMAD questionnaire, which offers a structured approach to examination of the four constructs that according to the NPT are central in implementation and normalisation processes. We aimed to evaluate the psychometric properties of the Swedish version S-NoMAD.

**Methods:**

Secondary analysis was performed on pooled S-NoMAD survey data from six implementation studies in different health and social care contexts. The NPT factor structure was tested by confirmatory factor analysis (CFA). Internal construct reliability was tested using Cronbach’s alpha. Validity was confirmed by assessing the fit of the CFA using the fit measures Comparative Fit Index, Tucker-Lewis Index, root mean square error of approximation and standardised root mean square residual. Pearson correlations amongst the latent construct and general questions about the intervention were calculated.

**Results:**

The estimation results of the CFA indicate that the four-factor model implied by the NPT fits the data reasonably well. The factor loadings are of good sizes and the fit indices do not imply a mis-specified model. A good internal construct validity, indicated by a good model fit to the NPT four-construct model and acceptable to good internal reliability, was shown. External validity was also demonstrated.

**Conclusions:**

The CFA results indicate that the S-NoMAD has good psychometric properties for capturing perceptions of people involved in various Swedish implementation studies conducted in both health and social care contexts, demonstrating its general applicability. They show that the S-NoMAD, unlike the majority of instruments for evaluation of implementation processes, is not context- and intervention-specific. The findings highlight the utility of the S-NoMAD and show that it meets some important criteria for pragmatic measures. Further studies are warranted on different interventions implemented in diverse contexts regarding the meaning of the magnitude of the NoMAD scores in order to clarify its potential value as a tool for assessment of implementation strategies and on psychometric properties beyond construct validity and internal construct reliability, for example on test–retest reliability and longitudinal studies focusing on responsiveness.

**Supplementary Information:**

The online version contains supplementary material available at 10.1186/s43058-025-00839-1.

Contributions to the literature

- The NoMAD tool offers a structured approach to evaluate the four specific constructs that according to the NPT are central in processes of implementation and normalisation of new interventions in care contexts.
- We carried out the first evaluation of psychometric properties of the Swedish version of the NoMAD (S-NoMAD), which includes data from both various health and social care contexts.
- A good internal construct validity of the S-NoMAD, indicated by a good model fit to the NPT four-construct model, was demonstrated for a mixed data set of six contextually distinct sub-cohorts
- The aggregated findings demonstrate external validity and support further usage of the S-NoMAD in both Swedish health and social care

## Introduction

Effective implementation of healthcare interventions is essential for improving patient outcomes, enhancing quality of care, and optimizing resource efficiency. However, the successful integration of complex interventions into existing practices is often challenging [1]. The development of valid and useful tools to better understand, evaluate and support the implementation processes in diverse settings is one way to advance the field of implementation science, and subsequently also the implementation in clinical practice [2]. One advocated avenue for development in the field is the use of relevant theory [3].

The Normalization Process Theory (NPT) is increasingly used for evaluating and understanding implementation processes of complex interventions in healthcare and other settings [4–8]. Central to the NPT is the concept of normalisation, which refers to the process by which new practices, technologies or ways of working become embedded and integrated into routine work processes [9]. One key tool for applying the NPT in research and practice is the Normalization Measure Development (NoMAD) questionnaire [10, 11]. Based on the NPT, the NoMAD questionnaire was developed to assess implementation processes from the perspective of individuals involved [10] and hence, ultimately to enable assessment of the normalisation of a new practice over time [12]. By examining four specific constructs that according to the NPT are central in implementation and normalisation processes, the NoMAD tool offers a structured approach to evaluating the processes, identifying barriers and facilitators to implementation and informing strategies for sustainable change. These mechanisms are conceptualised as follows: coherence (the sense-making work done to understand the new practice), cognitive participation (the relational work done to engage people in the new practice), collective action (the operational work done to enact the new practice) and reflexive monitoring (the appraisal work done to assess the new practice) [13]. Additionally, it is possible to use the NPT for investigating the normalisation potential [14], which can be understood by assessing the factors that are known to affect the implementation process in a specific setting and by the readiness of actors in the work of implementing a new practice and accepting it.

Key strengths of the NoMAD include its underlying theoretical basis and the intended usability from the perspectives of staff involved in implementation of complex interventions in healthcare and other contexts [10]. The originators have suggested that the NoMAD should be viewed as a “pragmatic measure” [15] that balances psychometric considerations against the requirements of implementation in real-life contexts [10]. Still, validation of the NoMAD questionnaire is crucial for ensuring its reliability, validity and utility in assessing normalisation processes in diverse contexts. The original development process of the tool in the United Kingdom, including item generation, cognitive testing and psychometric validation through confirmatory factor analysis, [10, 11], concluded that the questionnaire had good face validity, construct validity and reliability (internal consistency). Subsequently, this initial work has entailed the validation of different language versions: initially the Swedish S-NoMAD [16] by members of our research team, followed by a Dutch [12], a Brazilian [17], a Chinese [18] and a German [19] version, as well as a complementary validation of the original English version [20]. In general, these validation studies showed acceptable validity and reliability, for example by confirming the factor structure of the NoMAD questionnaire, but they also underlined the need for further testing and reporting of psychometric properties. In the current study we aimed to evaluate the psychometric properties of the S-NoMAD using data from six implementation projects across different Swedish care contexts. Specifically, we evaluated the construct validity and assessed the internal construct reliability.

In a previous pilot study [16], satisfactory psychometric properties for the initial stage of translation and validation of the S-NoMAD were demonstrated, generating the conclusion that “the development of a highly valid and reliable instrument is an iterative process that requires many extensive tests and trials in different settings and populations.” Building on this in the current study, we aimed to evaluate the psychometric properties of the S-NoMAD using pooled data from six implementation projects for several different implementation interventions targeting different study populations across different Swedish care contexts. Specifically, we evaluated the construct validity and assessed the internal construct reliability.

Furthermore, since data were included from six different real implementation projects in clearly different contexts, external validity will also be demonstrated.

## Methods

### Design

A psychometric evaluation study based on secondary analysis of S-NoMAD baseline data from six studies on implementation of different care interventions in diverse health and social care contexts. The COSMIN checklist [21] was used only sparingly as a guide (when appropriate) to enhance the clarity of reporting.

### The NoMAD structure and scoring

The NoMAD is a 23-item questionnaire divided into sections A–C, with section A consisting of two questions about the respondent, followed by section B with three general questions about the intervention to be implemented [22]. Section C contains 20 specific questions about the intervention, corresponding to the four constructs of the NPT, which have formally been defined by Finch et al. [10], and is therefore the main focus of the current study. The items of section C are Coherence and Cognitive Participation, which have four items each, Collective Action which has seven items and Reflexive Monitoring which has five. The items in section B are answered on a 10-point Likert scale ranging from ‘Not at all’ to ‘Completely’. The items in section C are answered using a 5-point Likert scale, ranging from ‘Disagree Strongly’ to ‘Agree Strongly’. In addition to the 5-point Likert scale, answers to questions in section C of the NoMAD questionnaire include three options where participants can select whether a particular statement is irrelevant for their role, irrelevant at this stage of the implementation or irrelevant for the intervention being implemented. A website [13] provides a users’ guide to the NPT and a presentation of the original NoMAD, including guidance for how the NoMAD can be used for implementation studies.

### Data collection

The data provided have been collected in different Swedish care contexts within six implementation projects in which the S-NoMAD questionnaire was used. Data collection (see below) was performed using an online survey (sub-study 4 and 6) or via paper–pencil (sub-studies 1–3 and 5). A total of *n* = 448 participants answered the survey, while after row-wise deletion due to non-response a total of 333 observations were left and used for the psychometric analyses. Although some of these projects were longitudinal, only baseline data (Table 1) was used for the analyses. The projects from which the data were obtained are briefly presented below:

- Study 1: *Implementing person-centred care: multiple case studies of strategies, leadership and health economy using process evaluation (IMPROVE I)*
  The research aim of the S-NoMAD part of the study was to investigate the extent to which healthcare staff perceived that person-centred care had become integrated into their way of working. The study was designed as an observational case study with repeated measurements. The intervention being implemented was person-centred care in the context of various healthcare units (in-patient geriatric and psychiatric care, outpatient nephrology care and primary care). The first set of questionnaires was distributed in 2018 to health professionals involved in clinical work at the included units. A total of 164 questionnaires were returned (positions: registered nurses *n* = 72, practical nurses *n* = 32, other *n* = 56, information missing *n* = 4; sex: female *n* = 142; age: > 40 years *n* = 105).
- Study 2: *Facilitation as a strategy to support the operationalisation and implementation of person-centeredness in ambulance care (IMPROVE 2)*
  In this study, designed as an intervention study with a pretest–posttest design, the training of facilitators as a strategy to support the implementation of person-centred care in the ambulance service was evaluated. The S-NoMAD was used to evaluate to what extent person-centred care was integrated into the ambulance personnel’s daily practice. Questionnaires were distributed to ambulance personnel at the four included ambulance stations on three occasions between 2019 and 2021. The first data collection was conducted approximately two months after the initiation of the implementation. At baseline, 81 questionnaires were returned.
- Study 3: *Establishment of long-term support after acquired brain injury: implementation and experiences of a community support network (BrainS)*
  In this observational study the implementation of practices related to a community support network for people with acquired brain injuries was explored. The S-NoMAD was used to investigate the normalisation process of the practices related to the community network among employees of the 15 actors (specialised rehabilitation services, social care services and patient organisations) involved in the network. The network was initiated in 2016 and successively developed and implemented with no influence from researchers. The S-NoMAD was distributed in 2018. A total of 37 questionnaires were returned.
- Study 4: *Implementation of internet-based cognitive behavioural therapy in primary care (iPriC)*
  An investigation of organisational models and their impact on outcome according to the Reach, Effectiveness, Adoption, Implementation and Maintenance (RE-AIM) framework was conducted and aimed to expand understanding of internet-based cognitive behaviour therapy (iCBT) implementation in primary care and to evaluate how different organizational models affected implementation outcomes using the RE-AIM framework. Research questions included identifying factors influencing iCBT implementation and assessing the impact of various organisational models on implementation outcomes, including RE-AIM. The study employed a mixed-methods research design to investigate factors influencing iCBT implementation across multiple levels involving patients, therapists and managers. Data have previously been analysed through thematic analysis and statistical tests over a two-year period [23]. A total of 53 iCBT therapists and 50 primary care managers completed the S-NoMAD surveys.
- Study 5: *Together for every child (TFVB)*
  A pilot study aimed to evaluate the implementation of a collaboration model between schools and social services within the municipalities and children’s primary and specialist care within the region’s health services [24]. Research questions included identifying factors influencing the implementation of the working model TFVB, in which the S-NoMAD survey was used along with semi-structured interviews. The design was pretest–posttest and mixed methods were used. Twenty of the participants (*n* = 20) in period 1 (June 2019) were managers and facilitators while the remaining did not answer or answered that they had another work position.
- Study 6: *The Family Talk Intervention in clinical practice when a parent with dependent children or a child is severely ill: An effectiveness-implementation study (FTI)*
  This study involved a psychosocial family-based intervention, the Family Talk Intervention (FTI), when a parent with dependent children or a child is severely ill with a life-threatening or life-limiting illness. This study aimed to evaluate the effects and the implementation process of FTI in three different clinical contexts where a parent with dependent children or a child has a life-threatening/life-limiting illness. An effectiveness-implementation hybrid design [25] with mixed methods was used. FTI education and training of social workers were conducted in 2021. After FTI education the social workers started using FTI in their everyday clinical practice. To examine the implementation process all social workers who were educated and trained in FTI answered internet-based surveys (including the S-NoMAD) directly after their FTI education (*n* = 46).

**Table 1:** Overview of the projects providing baseline data

*Sub Project/Study title*	*Design*	*Intervention*	*Context/Setting*	*S-NoMAD baseline n*	*Used in analysis*
1. Implementing person-centred care: multiple case studies of strategies, leadership and health economy using process evaluation (IMPROVE 1)	Observational case study	Person-centred care	Hospital care, psychiatric care, primary care	Baseline: *n* = 164	118
2. Facilitation as a strategy to support operationalization and implementation of person-centeredness in ambulance care (IMPROVE 2)	Intervention study with pre-post design	Person-centred care	Ambulance care	Baseline: *n* = 81	75
3. Establishment of long-term support after acquired brain injury – implementation and experiences (BrainS)	Cross sectional	Practices related to the community network	Health care, social care, patient organisations	37	16
4. The influence of organizational models on the implementation of internet-based cognitive behaviour therapy in primary care (iPriC)	Cross-sectional	Internet-based cognitive behaviour therapy	Primary care	102	75
5. Together for every child (TFVB)	Pre-post design	Together for every child	Municipality	Baseline: *n* = 24	17
6. The Family Talk Intervention in clinical practice (FTI)	Cluster-randomized trial, pre-post design	A psychosocial family-centred intervention	Specialized palliative home care, paediatric hospital care, cancer hospital care	Baseline: *n* = 40	32

The column *Used in analysis* corresponds to the number of complete cases

### Data analysis

#### Psychometric analyses

Construct validity was examined by confirmatory factor analysis (CFA) [26] and was used to confirm the S-NoMAD structure based on the NPT theory, that is, its four-factor structure. Estimation and analysis were done in R [27], with the packages lavaan [28] and semTools [29]. As the items were measured on a five-ordinal scale (strongly agree; agree; neither agree nor disagree; disagree; strongly disagree) the diagonally weighted least squares with robust standard errors (WLSMV) option and ordered = TRUE were used when estimating the model [10].

The implementation in R requires complete cases, hence row-wise deletion was carried out. Table 1 displays the number of observations of the full sample and after row-wise deletion. A sensitivity analysis was carried out using the default estimation method for CFA with continuous responses, first with all observations and then with those remaining after row-wise deletion. Factor loading close to zero or relatively large indicates a misspecified model. Model fit was assessed using the comparative fit index (CFI), the Tucker-Lewis index (TLI), the root mean square error of approximation (RMSEA) and the standardised root mean square residual (SRMR). Additionally, the ratio between the ${\chi }^{2}$ and the degrees of freedom was considered. There are no strict critical values for these fit indices. Usually, good values of the CFI and TLI are values above 0.90, while RMSEA and SRMR should preferably be less than 0.1 or even as low as 0.05 [30]. An ${\chi }^{2}$ degrees of freedom ratio below 5 can be seen as reasonable [31]. Reliability was measured by the ordinal version of Cronbach alpha where a value above 0.6 is usually considered reliable [32].

In addition to the four-factor model, a one-factor model intended to be interpreted as a construct of an overall normalisation factor was estimated. The estimated construct from the one-factor model was labelled the normalisation score. All items were loaded on this factor [10]. The estimation of correlations, factor score and so forth followed the methodology as outlined above. The one-factor model is a restricted four-factor model. Hence, a statistical likelihood ratio test was conducted with the one-factor model as null and the four-factor model as an alternative in order to statistically test whether a one-factor model is sufficient to describe the data. The correlations between the latent variables were Pearson correlations on estimated factor scores. The factor scores were estimated using the Empirical Bayes modal method as implemented in lavaan. Correlations (in absolute value) between 0 and 0.3 are often denoted as weak, between 0.3 and 0.7 as moderate and above 0.7 as a strong linear relationship [33].

## Results

### Descriptive data

Six implementation projects across different care contexts contributed data for the current study. An overview of the included studies, including their design and context, the total number of answered baseline questionnaires per study and the number (reduced due to the requirement for use of R) analysed is presented in Table 1.

Response to section A showed that respondents represented a variety of different professional roles in relation to the interventions that were being implemented. Professions reported were, for example, physician, nurse, assistant nurse, physiotherapist, occupational therapist, emergency medical technician, manager, psychologist, speech therapist, job coach and social worker. The variation was also large in terms of number of years worked within the organisations where the implementation took place, with a range of less than 1 to 34 years. Answers from section B were judged less relevant for the current study and were not analysed.

Row-wise deletion due to non-response, multiple response or response with not-relevant option in (parts of) section C concerns 115 respondents (Supplementary Table 1). The sensitivity analysis using the default estimation method for CFA with continuous responses, first with all observations and then with those remaining after row-wise deletion showed minor differences implying no systematic patterns in the non-response.

For 2.6% of the total answers, participants chose one of the three ‘irrelevant’ options, and less than 1% of the data were missing due to non-response or multiple response. Notably, many participants were unable to answer question C4.3, “I value the effects that the intervention has had on my work” using the Likert scale. The “Irrelevant for my role” option for this question was chosen by 4.7% of participants, and “Irrelevant at this stage” by 2.2%. Missing data were most frequent in the last part (C.4) of section C, but even there accounted for less than 2% of the total responses (Supplementary Table 1 and Fig. 1). Only in study 3 it caused substantial loss of data.

Estimation results for the factor loadings from the confirmatory factor analysis of the four- factor model can be found in Table 2. All are statistically significant at any reasonable level and are around 0.4 to about 0.9, except for one which is negative (−0.27). The fit indices are shown in Table 3 where CFI and TLI are higher than required while RMSEA and RMSR are somewhat higher than the common rules of thumb. The syntax for estimation, the polychoric corelations and the thresholds are given in the supplementary files.

**Table 2:** Confirmatory factor analysis (CFA) and Factor loadings for the latent constructs from the Normalization process theory (NPT) four-factor model (columns 1–4) and for the one-factor model (last column)

Item	Coherence	Cognitive Participation	Collective Action	Reflexive Monitoring	Normalization score*
I can see how [the intervention] differs from usual ways of working	0.66				0.59
Staff in this organisation have a shared understanding of the purpose of [the intervention]	0.44				0.40
I understand how [the intervention] affects the nature of my own work	0.76				0.68
I can see the potential value of [the intervention] for my work	0.91				0.80
There are key people who drive [the intervention] forward and get others involved		0.43			0.39
I believe that participating in [the intervention] is a legitimate part of my role		0.76			0.69
I’m open to working with colleagues in new ways to use [the intervention]		0.88			0.84
I will continue to support [the intervention]		0.96			0.91
I can easily integrate [the intervention] into my existing work			0.66		0.53
[The intervention] disrupts working relationships			−0.27		−0.23
I have confidence in other people’s ability to use [the intervention]			0.57		0.45
Work is assigned to those with skills appropriate to [the intervention]			0.41		0.30
Sufficient training is provided to enable staff to implement [the intervention]			0.72		0.55
Sufficient resources are available to support [the intervention]			0.66		0.50
Management adequately supports [the intervention]			0.60		0.47
I am aware of reports about the effects of [the intervention]				0.48	0.46
The staff agree that [the intervention] is worthwhile				0.64	0.61
I value the effects that [the intervention] has had on my work				0.70	0.67
Feedback about [the intervention] can be used to improve it in the future				0.70	0.67
I can modify how I work with [the intervention]				0.59	0.56

All significant at the 0.01 level

^*^Normalization Score = The estimated overall construct from the one-factor model

**Table 3:** Results from analysis of how well the model fits data using fit indices

	**rmsea**	**cfi**	**tli**	**srmr**	${\chi }^{2}$**/df**
ordinal cfa four-factors	0.10	0.96	0.95	0.09	4.34
ordinal cfa one-actor	0.14	0.92	0.91	0.11	7.12

*CFI* Comparative fit Index, *TLI* Tucker Lewis Index, *RMSEA* root mean square arror of Approximation, *SRMR* standardised root mean square residual, ${\chi }^{2}$/df is the ratio of ${\chi }^{2}$ divided by the degrees of freedom

The correlations amongst the latent factors were often high, ranging from about 0.56 to 0.99 (Table 4). The correlations between the latent factors and the general questions ranged from 0.3 to 0.7. The sensitivity analysis, which used standard methods for continuous data (not reported), shows that the full sample and the row-wise deleted sample yielded almost the same results. These results differ from the one used above, which is based on appropriate methods for ordinal data. The conclusion was that the difference is due to estimation method and not due to using row-wise deletion.

**Table 4:** Pearson correlations between latent constructs

	**Coherence**	**Cognitive Participation**	**Collective Action**	**Reflexive Monitoring**	Normalization score*
Coherence	1.00				
Cognitive Participation	0.82	1.00			
Collective Action	0.66	0.56	1.00		
Reflexive Monitoring	0.94	0.89	0.81	1.00	
Normalization score	0.93	0.94	0.77	0.99	1.00

All significant at the 0.01 level

^*^Normalization Score = The estimated overall construct from the one-factor model

The one-factor model has factor loadings similar to the four-factor model (Table 3), though slightly worse fit statistics. The fit statistics are 0.92 (CFI), 0.91 (TLI), 0.14 (RMSEA) and 0.11 (SRMR). The ratio of the ${\chi }^{2}$ and degrees of freedom is 7.12. The likelihood ratio test between the four-factor and the one-factor model rejects the one-factor model with a test statistic of 253.18 (${\chi }^{2}$ with 6 degrees of freedom, *p*-value < 0.001). Analysis of the internal construct reliability showed that the lowest Cronbach alpha was 0.63 (Collective Action) while the others are 0.76 or higher (Table 5), all above the cut-off.

**Table 5:** Internal consistency measured by the Ordinal Cronbach alpha

	**Coherence**	**Cognitive Participation**	**Collective Action**	**Reflexive Monitoring**	Normalization score*
Cronbach alpha	0.76	0.81	0.63	0.75	0.88

The Cronbach alpha is the ordinal version of Zumbo et al. (2007)

^*^ Normalization Score = The estimated overall construct from the one-factor model

## Discussion

This is the first evaluation of the psychometric properties of the S-NoMAD that includes implementation study results from both health and social care contexts, obtained from several projects. The purpose was to evaluate the psychometric properties of the S-NoMAD based on the NPT, focusing on the 20 specific questions about the different interventions being implemented.

The mean response score across the data set (*n* = 333) used in psychometric analyses was 2.26, which is slightly below the midpoint of the range and is narrow, which may be explained by the fact that all data were collected at baseline for each of the sub-studies. Missing data and not-relevant options in responses were rather infrequent in our data set (Supplementary, Table 1 and Fig. 1).

Based on the NPT a four-factor model was specified and estimated. The results show that the estimated factor loadings are of appropriate sizes. All are statistically significant at any reasonable level and are around 0.4 up to about 0.9. This is important as factor loadings close to zero or very large would imply a different factor structure than the one identified. However, one is negative (−0.27) due to the fact that the question is phrased as a negatively valued statement instead of a positively valued one: “the intervention disrupts working relationships”. The discrepancy of this question and associated difficulties have also been noted in previous research. For example, in the original translation and pilot test study of the S-MoMAD [16], this question and two others were deleted from the analyses to achieve an acceptable model fit, which was judged to be caused by the linguistic interpretation and too small of a sample. Additionally, a Dutch NoMAD study [12] identified the same question as falling below the validation standard. A reasonable explanation for the similar findings in both of these studies can be that the term “disrupt” has a more negative meaning among both the Swedish and the Dutch respondents compared to English speaking participants. Deleting this question may therefore be considered. Though, as highlighted by Batterham et al., such a change would only be based on empirical statistical associations, while “construction of and changes to measurement instruments need to conceptually make sense and have solid evidence that the benefits of changing a validated measure outweigh the risks, such as problems with inconsistent scale versions” [34] (page 7).

The current evaluation of fit to the NPT factor model nevertheless shows a good agreement of the NPT constructs. This is confirmed by the fit measures CFI and TLI, which are higher than required and indicate a good fit. RMSEA and RMSR, on the other hand, indicate a worse fit than CFI and TLI with values around the critical 0.1, though are not alarmingly large. The ${\chi }^{2}$/degrees of freedoms ratio is reasonable as it is below the commonly used rule of thumb, namely 5. Overall, it is fair to conclude that the estimated model shows good internal validity.

Turning to internal reliability, Cronbach alpha results were from 0.63 for the factor Collective Action up to 0.81 for the factor Cognitive Participation. This implies that we are reasonably sure that we have estimated the latent factors sufficiently well.

The correlation among some of the latent variables is rather high (Table 2). The correlations range from 0.56 to 0.94, the higher between Coherence and Reflexive Monitoring. Further, Coherence and Cognitive Participation have high correlations, 0.93 and 0.94 respectively. These correlations are higher than, for example, those found by Finch et al. (2018). Turning to the correlations between the constructs and general normalisation assessment items, we found that they are about −0.3 to −0.6.

The high correlations amongst the latent factors suggest that a one-factor model could be appropriate. The general pattern of the estimated factor loadings is similar to the four-factor, though often somewhat smaller. This is true in general but not for the factor loadings for the Reflexive Monitor factor, where the one-factor loadings are slightly larger. This is an indication of worse fit. This remark is supported by the fact that the fit indices CFI, TLI, RMSEA and RMSR are all worse in the one-factor model. The statistical test does not favour the one-factor model since it was shown to be too restrictive compared to the four-factor model. Statistical tests have been used to test if it is possible to reduce the number of factors from four to three by, e.g., imposing the restriction that Coherence and Reflexive Monitoring is the same factor. We reject all such tests implying that there are strong indications of a four factor model. The correlations between the factor scores from the four-factor model and the one-factor model show an extremely high correlation of 0.99 between Reflexive Monitoring and the normalisation score. A reasonable interpretation is that the one-factor model is dominated by that factor. This is supported by a Cronbach alpha of 0.88, which is the best value of all factors. This signifies the importance of estimating a four-factor model as the one-factor cannot capture all relevant aspects of the NPT. When the instrument is used it should be interpreted with the understanding that each factor represents a distinct aspect of the NPT, and that all four factors together provide a more nuanced and complete picture of the theory's application. It is therefore important not to simplify the instrument by reducing it to a single factor, as this could lead to overlooking or misunderstanding important aspects of the theory. A four-factor model allows for a more detailed and accurate assessment of the NPT in the contexts where the instrument is applied. Overall, the results in the current multicentre study confirm the results from the previous pilot study [16]. Moreover, based on the utilization of different populations in different contexts, good external validity was demonstrated for S-NoMAD usage in Swedish both health and social care.

The current study has limitations that should be considered, which implies that the main drawback is the large correlation amongst the latent factors. It is difficult to know if this is a defiance of the survey, that is, not being able to separate the concepts, or if this is just bad luck with the sample. As we have not seen this in other studies using the NoMAD it might also be due to the translation. A strength of our methodology, on the other hand, is the inclusion of data from six implementation studies conducted in different contexts, including healthcare and social services. This dataset was sufficiently large to enable adequate analyses and showed a good model fit. The obtained results showed that both the S-NoMAD questionnaire and the NPT have sufficient robustness for the evaluation of implementation mechanisms in culturally different care contexts. The findings thus show that the S-NoMAD, unlike the majority of instruments for evaluation of implementation processes [35], is not context- and intervention-specific, but can be useful for evaluation of different implementation processes in Swedish health and social care.

## Conclusions

The results of the CFA indicate that the S-NoMAD has good psychometric properties for capturing perceptions of people involved at baseline in various Swedish implementation studies conducted in both health and social care contexts, demonstrating its general applicability and external validity. A good internal construct validity indicated by a good model fit to the NPT four-construct model and acceptable to good internal reliability was also shown. However, the latter are within the range that can be expected and raise no questions about reliability.

These findings highlight the utility of the S-NoMAD and show that it meets some important criteria for pragmatic measures. Nevertheless, further studies are warranted on different interventions implemented in diverse contexts, regarding the significance of the magnitude of the NoMAD scores as a whole as well as distributed among its subscales, in order to clarify its potential value as a tool for assessment and guidance of implementation and normalisation strategies. The same applies to the need for future studies on psychometric properties of the S-NoMAD beyond construct validity and internal construct reliability on, for example, test–retest reliability and longitudinal studies focusing on responsiveness.

## Supplementary Information

Supplementary Material 1.

## Data Availability

The material analysed during the current study is not publicly available due to its content of personal data. Datasets generated may be available from the principal investigator Anna Cristina Åberg upon reasonable request, after ethical considerations.

## Abbreviations

CFA: Confirmatory Factor Analysis
CFI: Comparative Fit Index
FTI: Family Talk Intervention
iCBT: Internet-based Cognitive Behaviour Therapy
NPT: Normalization Process Theory
RE-AIM: Reach, Effectiveness, Adoption, Implementation and Maintenance
RMSEA: Root Mean Square Error of Approximation
S-NoMAD: The Swedish version of the NoMAD questionnaire
SRMR: Standardised Root Mean Square Residual
TLI: Tucker-Lewis Index
WLSMV: Diagonally weighted least squares with robust standard errors
