# Supplementary material for: A multicentre validation study of the Swedish version of the Normalization Process Theory Measure S-NoMAD
Source: Implement Sci Commun. 2025 Dec 15;7:11. doi: 10.1186/s43058-025-00839-1 (PMC12838009; doi:10.1186/s43058-025-00839-1)
Supplement: Supplementary file 1 — Supplementary Material 1. [file 43058_2025_839_MOESM1_ESM.docx]

**Supplementary material**

**Table 1.** Overview of the not-relevant (N.R) options and missing answers in section C of the NoMAD questionnaire. Results are provided as percentages of the total number of possible responses.

|  | Question | N.R. for my role | N.R. at  this stage | N.R. for the intervention | Missing data* |
| --- | --- | --- | --- | --- | --- |
| C.1.1 | I can see how [the intervention] differs from usual ways of working | 2.5 | 0.7 | 0.7 | 1.3 |
| C.1.2 | Staff in this organisation have a shared understanding of the purpose of [the intervention] | 1.8 | 0.2 | 0 | 0.7 |
| C.1.3 | I understand how [the intervention] affects the nature of my own work | 3.3 | 0.4 | 0 | 0.7 |
| C.1.4 | I can see the potential value of [the intervention] for my work | 1.1 | 0.2 | 0.2 | 0.7 |
| C.2.1 | There are key people who drive [the intervention] forward and get others involved | 0.4 | 0.7 | 0.2 | 0 |
| C2.2. | I believe that participating in [the intervention] is a legitimate part of my role | 1.6 | 0.2 | 0.2 | 0 |
| C.2.3 | I’m open to working with colleagues in new ways to use [the intervention] | 1.8 | 0.2 | 0.4 | 0 |
| C.2.4 | I will continue to support [the intervention] | 0.4 | 0.2 | 0.2 | 0 |
| C.3.1 | I can easily integrate [the intervention] into my existing work | 1.3 | 0.7 | 0.2 | 0.2 |
| C.3.2 | [The intervention] disrupts working relationships | 2.2 | 0.7 | 1.1 | 0.7 |
| C.3.3 | I have confidence in other people’s ability to use [the intervention] | 0.7 | 0.2 | 0 | 0.7 |
| C.3.4 | Work is assigned to those with skills appropriate to [the intervention] | 1.6 | 0.4 | 0.4 | 0.9 |
| C.3.5 | Sufficient training is provided to enable staff to implement [the intervention] | 3.1 | 0.9 | 0.2 | 0.2 |
| C.3.6 | Sufficient resources are available to support [the intervention] | 1.6 | 0.9 | 0.4 | 0.4 |
| C.3.7 | Management adequately supports [the intervention] | 0.2 | 0 | 0 | 1.1 |
| C.4.1 | I am aware of reports about the effects of [the intervention] | 2.0 | 0.9 | 0.9 | 1.6 |
| C.4.2 | The staff agree that [the intervention] is worthwhile | 1.3 | 0.2 | 0 | 1.8 |
| C.4.3 | I value the effects that [the intervention] has had on my work | 4.7 | 2.2 | 0.2 | 1.3 |
| C.4.4 | Feedback about [the intervention] can be used to improve it in the future | 0.9 | 0.9 | 0.2 | 1.8 |
| C.4.5 | I can modify how I work with [the intervention] | 2.2 | 0.2 | 0 | 1.6 |

* Due to non-response or multiple response

**
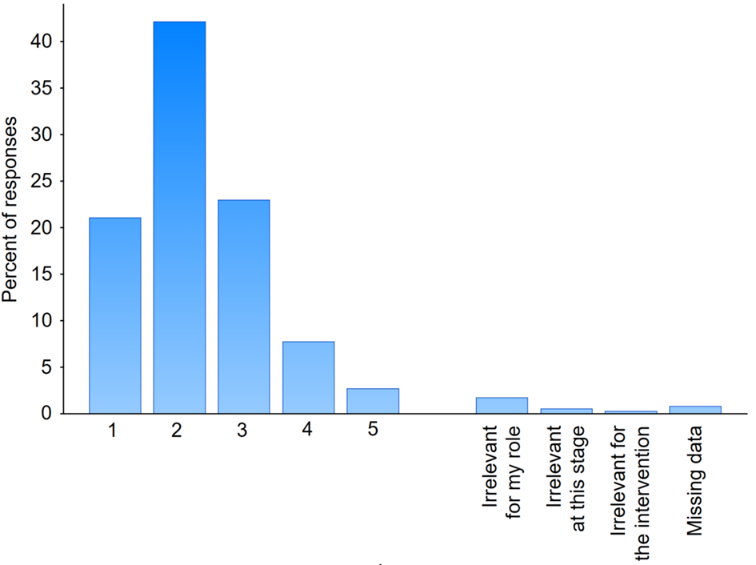
**

**Figure 1. Distribution of scores for the part C of S-NoMAD questionnaire (n=333)**
